# Incidence and health burden of 20 rare neurological diseases in South China from 2016 to 2022: a hospital-based observational study

**DOI:** 10.1186/s13023-025-03704-5

**Published:** 2025-04-08

**Authors:** Jingjing Li, Shujin Tang, Jiaoxing Li, Xin Huang, Yu Liu, Jinsheng Zeng, Yuhua Fan

**Affiliations:** 1 Department of Neurology, The First Affiliated Hospital, Guangdong Provincial Key Laboratory of Diagnosis and Treatment of Major Neurological Diseases, National Key Clinical Department and Key Discipline of Neurology, Sun Yat-Sen university, No. 58 Zhongshan Road 2, Guangzhou, 510080 China; 2 School of Public Health and Management, Guangzhou University of Chinese Medicine, Guangzhou, China

**Keywords:** Rare neurological disease, Health burden, Juvenile, China

## Abstract

**Background:**

Rare neurological diseases (RNDs) result in severe health burdens worldwide. Data from China are limited. We aimed to investigate the health burden of 20 RNDs in Guangdong Province (GD), which contains two-thirds of the population of South China.

**Methods:**

The hospitalization data of 20 RNDs were described using hospital-based front sheet data from 3,037 hospitals of GD from 2016 to 2022. The 20 RNDs included amyotrophic lateral sclerosis (ALS), Charcot-Marie-Tooth Disease, cerebral autosomal dominant arteriopathy with subcortical infarcts and leukoencephalopathy, congenital myotonia, congenital myasthenic syndrome, Dravet syndrome, Fabry disease, hereditary spastic paraplegia, Huntington disease, Leber hereditary optic neuropathy, mitochondrial encephalopathy (ME), multi-focal motor neuropathy, myotonic dystrophy, primary hereditary dystonia, progressive muscular dystrophy (PMD), spinal and bulbar muscular atrophy, spinal muscular atrophy (SMA), spinocerebellar ataxia, Wilson disease (WD) and X-linked adrenoleukodystrophy. Age were presented as mean and standard deviation while length of hospital stay as median and interquartile range (25th and 75th percentiles). The other variables were described as number and percentage. The data were analyzed by Joinpoint regression.

**Results:**

There were 9,351 cases, including 330 ICU and 155 death cases. The average age was 33.7 ± 22.0 y, and 63.8% of patients were male. From 2016 to 2022, the number of RND (and juvenile RND) cases were 1034 (184), 1174 (293), 1443 (374), 1422 (320), 1331 (337), 1432 (409) to 1515 (515). ICU (and juvenile ICU) cases rose from 28 (3), 34 (6), 24 (4), 38 (11), 46 (13), 54 (24) to 106 (56). Joinpoint regression showed significant upward trend in percentages of juvenile and juvenile ICU cases (APC = 8.13, *P*< 0.05; APC = 28.42, *P*< 0.05). The fop five RNDs were WD, ASL, PMD, ME, and SMA, which accounted for 79.7% of all, 99.1% of ICU, and 94.8% of death cases.

**Conclusions:**

We demonstrated that the increase in health burden of RNDs was mainly evident in juveniles in South China from 2016 to 2022. The top 5 RNDs accounted for majority of the critical patients.

**Supplementary Information:**

The online version contains supplementary material available at 10.1186/s13023-025-03704-5.

## Introduction

Rare diseases (RDs), also known as orphan diseases, are any diseases or conditions with a low prevalence, and they are often debilitating or even life threatening. There are an estimated 5,000–8,000 RDs, many of which have neurological manifestations [1, 2]. There are at least 90 million people with RDs in China [3]. Although they are called rare, RDs are surprisingly common in China, where they create a huge healthcare and economic burden [4]. Rare neurological diseases (RNDs) constitute a significant proportion of RDs; almost 50% of all RDs affect the nervous system and muscles [5, 6]. One-third of the 121 RDs listed in the Chinese First National List of RDs are RNDs [7]. The prevalence of RNDs has been estimated at between 8.9% and 53.4%, according to studies from different countries and different populations [8, 9]. Except in Hong Kong, few studies have investigated RNDs comprehensively in China [10, 11]. Most of the research in China has been on non-neurological RDs, with only a few studies focusing on RNDs [3, 12, 13, 14, 15].

Guangdong Province (GD), located in South China, has a population of 126.84 million; thus, it represents the province with largest proportion (8.93%) of the Chinese population and accounts for two-thirds of the population of South China [16]. Based on a study by the Beijing Society of RDs using hospital record front sheets for 15 million hospitalizations from 2014 to 2015, GD had the third highest number of RDs in China (after Beijing and Shanghai) [17]. However, there is no data on RNDs in GD. Therefore, we aimed to analyze the front sheet data for patients with 20 RNDs recorded in the Chinese First National List of RDs from 2016 to 2022 taken from the direct reporting system for health information in GD. We aimed to estimate the health burden of RNDs in South China over the 7-year period.

## Methods

This retrospective study was approved by the institutional review board of the First Affiliated Hospital of Sun Yat-sen University (No. [2024]035). Data were extracted from the direct reporting system for health information in GD. The system includes a collection of front sheets from the hospitalization medical records of all discharged patients in GD. We collected 20 degenerative or genetic RNDs of the Chinese First National List. They included amyotrophic lateral sclerosis (ALS), Charcot-Marie-Tooth Disease (CMT), Cerebral autosomal dominant arteriopathy with subcortical infarcts and leukoencephalopathy (CADASIL), congenital myotonia (CM), Congenital myasthenic syndrome (CMS), Fabry disease, Hereditary spastic paraplegia (HSP), Huntington disease (HD), Leber hereditary optic neuropathy (LHON), Myotonic dystrophy (MD), Mitochondrial encephalopathy (ME), Multi-focal motor neuropathy (MMN), Primary hereditary dystonia (PHD), Progressive muscular dystrophy (PMD), Dravet syndrome, Spinal and bulbar muscular atrophy (SMA), Spinal muscular atrophy (SMA), spinocerebellar ataxia (SCA), Wilson disease (WD) and X-linked adrenoleukodystrophy. We must declare that ME and PMD are categories of rare diseases containing groups of precise diseases. We used these entities in this article to be consistent with the 121 rare diseases listed in China’s First List of Rare Diseases. We searched for RNDs in the system according to the codes in the 10th Revision of the International Classification of Diseases (ICD-10). Based on the patient’s name and ID number, “the same person” tag is generated within the database of our study. If a patient has multiple discharges within the same year, these are counted as one case of RND. The number of all discharged cases in GD was 15.446 million, 16.325 million,17.088 million, 18.145 million, 15.675 million, 17.300 million and 17.526 million, over the 7-year period. (https://www.gdhealth.net.cn/html/2024/tongjishuju1_0514/4402.html)

The Chinese First National List of RDs, which was published in 2018 [7], has 121 listed RDs, nearly one-thirds of which are RNDs. Our study collected data on 20 degenerative or genetic RNDs in the Chinese First National List. We excluded metabolic and autoimmune RNDs as well as systemic RDs which involved neurological manifestations. Demographic information included average age, gender, admission hospital, on-admission department, number of ICU cases, number of deaths, percentage of different payments (urban employee basic medical insurance, UEBMI; urban resident basic medical insurance, URBMI; new rural cooperative system, NRCS; free medical service, FMS; social insurance and others), and average length of stay. The top 5 RNDs with the most cases were selected and analyzed separately. Juveniles are defined as individuals from birth to 18 years of age. Standardized front sheet was supplied in the supplementary data.

### Statistical analysis

Age was presented as mean and standard deviation while length of hospital stay as median and interquartile range (25th and 75th percentiles). The other variables were described as number and percentage. Joinpoint regression models [18] were used to examine the temporal trends of proportional indicators (RNDs in discharged patients, juveniles in discharged patients with RNDs, ICU cases in discharged patients with RNDs, juveniles in ICU patients with RNDs) during 2016–2022. The Joinpoint Regression program (version 5.0.2; National Cancer Institute, Calverton, MD, USA) was utilized to estimate their evolving patterns in a structured manner and to test the statistical significance between joinpoints. A maximum number of three-line segments (two joinpoints) were established in the models. The annual percent change (APC) was calculated to indicate the direction and magnitude of the trends. *P* value less than 0.05 was considered statistically significant.

## Results

### General description of patients with 20 RNDs

**Fig. 1:**
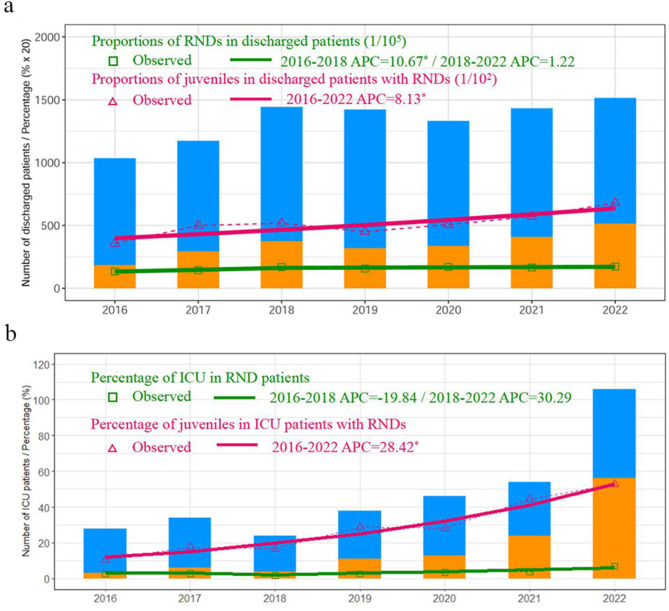
The number of inpatient juvenile cases and juvenile intensive care unit(ICU) patients of 20 rare neurological diseases increased annually. (a) Proportions of RNDs in discharged patients and proportions of juveniles in discharged patients with RNDs. The number of 20 RNDs were 1034, 1174, 1443, 1422, 1331, 1432 to 1515 from 2016 to 2022 while that of all discharged cases were 15.446 million, 16.325 million,17.088 million, 18.145 million, 15.675 million, 17.300 million and 17.526 million, over the 7-year period in Guangdong; (b) Percentage of ICU in RND patients and percentage of juveniles in ICU patients with RNDs. There were 28 ICU cases in 2016, 34 cases in 2017, 24 cases in 2018, 38 cases in 2019, 46 cases in 2020, 54 cases in 2021 and 106 cases in 2022. The percentage of juveniles among these ICU patients grew from 10.71% (3 cases), 17.65% (6 cases), 16.67% (4 cases), 28.95% (11 cases), 28.26% (13 cases), 44.44% (24 cases) to 52.83% (56 cases) in the 7-year period

There were 9,351 cases of the 20 degenerative or genetic RNDs recorded in the direct reporting system for health information from 3037 hospitals in GD from 2016 to 2022, of which 63.8% were male (*n* = 5964) and 36.2% (*n* = 3378) were female patients. The average age was 33.7 ± 22.0 y. The number of 20 RNDs increased almost annually, from 1034, 1174, 1443, 1422, 1331, 1432 to 1515 from 2016 to 2022. The percentage of juveniles increased from 17.8%, 25.0%, 25.9%, 22.5%, 25.3%, 28.6 to 34.0% year by year. Joinpoint regression demonstrated a slow but insignificant increase on proportions of RNDs in discharged patients during 2018 and 2022 (APC = 1.22 with a unit of 1/10, *P*>0.05, Fig. 1A) even though a short-term growth between 2016 and 2018 was observed (APC = 10.67, *P*<0.05, Fig. 1A). Meanwhile, the increase of inpatient juvenile RND was significant in the 7-year period (APC = 8.13 with a unit of 1/10^2^, *P*<0.05, Fig. 1A). The general characteristics of the 20 RNDs are summarized separately in Table 1. The number of RNDs, ICU RNDs, and juvenile RNDs for each of the 20 diseases from 2016 to 2022 were presented in supplementary data. (Supplementary Table 1, Tables 2, and Table 3). The age distribution of RNDs and ICU RNDs in these 7 years were presented in Supplementary Tables 4 and Table 5.

**Table 1:** The hospitalization data of the 20 degenerative or genetic rare neurological diseases admitted to hospital in Guangdong province of China (2016–2022)

	2016	2017	2018	2019	2020	2021	2022	Total
Number of patients	1034	1174	1443	1422	1331	1432	1515	9351
Age, mean (SD), y	33.1 (21.5)	32.2 (21.6)	33.5 (21.2)	36.7 (21.7)	35.4 (22.7)	34.8 (22.1)	30.8 (22.2)	33.7 (22.0)
Sex (male), n (%)	669 (64.7)	736 (62.7)	916 (63.5)	879 (61.8)	890 (66.9)	901 (62.9)	973 (64.2)	5964 (63.8)
Juveniles, n (%)	184 (17.8)	293 (25.0)	374 (25.9)	320 (22.5)	337 (25.3)	409 (28.6)	515 (34.0)	2432 (26.0)
Juveniles (male), n (%)	129 (70.1)	196 (66.9)	255 (68.2)	218 (68.1)	247 (73.3)	291 (71.1)	346 (67.2)	1682 (69.2)
ICU patients, n (%)	28 (2.7)	34 (2.9)	24 (1.7)	38 (2.7)	46 (3.5)	54 (3.8)	106 (7.0)	330 (3.5)
Juvenile ICU patients, n (%)	3 (10.7)	6 (17.7)	4 (16.7)	11 (29.0)	13 (28.3)	24 (44.4)	56 (52.8)	117 (35.5)
Length of hospital stay, median (IQR)	11 (6–18)	10 (6–15)	10 (6–14)	9 (6–14)	9 (5–14)	9 (5–14)	6 (2–12)	9 (5–14)
Hospital admission	1380	1552	1976	1998	1814	2093	2865	13,678
Admission department, n (%)								
Neurology	720 (52.2)	727(46.8)	995 (50.4)	997 (50.0)	789 (43.5)	895 (42.8)	1072 (37.4)	6195 (45.3)
General medicine	288 (20.9)	318(20.5)	397 (20.1)	416 (20.8)	359 (19.8)	424 (20.3)	626 (21.8)	2828 (20.7)
Pediatrics	95 (6.9)	181(11.7)	216 (10.9)	172 (8.6)	257 (14.2)	278 (13.3)	615 (21.5)	1814 (13.3)
Others	277 (20.1)	326(21.0)	368 (18.6)	413 (20.7)	409 (22.5)	496 (23.7)	552 (19.3)	2841 (20.7)
Source of patients n (%)								
District of the hospital	380 (27.5)	426 (27.4)	560 (28.3)	612 (30.6)	590 (32.5)	577 (27.6)	818 (28.6)	3963 (29.0)
The other district of local city	275 (20.0)	310 (20.0)	388 (19.6)	417 (20.9)	396 (21.8)	579 (27.7)	997 (34.8)	3362 (24.6)
The other city of Guangdong province	501 (36.3)	561 (36.1)	660 (33.4)	625 (31.3)	569 (31.4)	662 (31.6)	789 (27.5)	4367 (31.9)
The other province	220 (15.9)	249 (16.0)	355 (18.0)	332 (16.6)	251 (13.8)	266 (12.7)	250 (8.7)	1923 (14.1)
Others	4 (0.3)	6 (0.4)	13 (0.7)	12 (0.6)	8 (0.4)	9 (0.4)	11 (0.4)	63 (0.4)
Payment methods, n (%)								
UEBMI	423 (30.7)	528 (34.0)	651(32.9)	741 (37.1)	607 (33.5)	806 (38.5)	1117 (39.0)	4873 (35.6)
URBMI	262 (19.0)	292 (18.8)	447 (22.6)	433 (21.7)	400 (22.1)	421 (20.1)	635 (22.2)	2890 (21.1)
NRCS	153 (11.1)	116 (7.4)	101 (5.1)	119 (6.0)	88 (4.9)	66 (3.2)	72 (2.5)	715 (5.2)
FMS	0 (0)	0 (0)	4 (0.2)	1 (0.1)	1 (0.05)	0 (0)	0 (0)	6 (0.04)
fully self-funded	402 (29.1)	454 (29.4)	491 (24.8)	380 (19.0)	301 (16.6)	349 (16.7)	251 (8.8)	2628 (19.2)
poverty relief	7 (5.1)	28 (1.8)	44 (22.3)	41 (2.1)	26 (1.4)	21 (1.0)	36 (1.3)	203 (1.5)
social insurance	111 (8.0)	106 (6.8)	199 (10.1)	227 (11.4)	238 (13.1)	249 (11.9)	268 (9.4)	1398 (10.2)
Other	22 (2.6)	28 (1.8)	39 (2.0)	56 (2.8)	153 (8.4)	181 (8.6)	486 (17.0)	965 (7.1)

SD, standard deviation; IQR, interquartile range; ICU, intensive care unit; UEBMI, urban employee basic medical insurance; URBMI, urban resident basic medical insurance; NRCS, new rural cooperative system; FMS, free medical service

**Table 2:** The hospitalization data of the death cases of the 20 degenerative or genetic rare neurological diseases admitted to hospital in Guangdong Province of China (2016–2022)

	2016	2017	2018	2019	2020	2021	2022	Total
n	14	27	23	24	25	17	25	155
Percentage, (%)	1.4 (14/1034)	2.3 (27/1174)	1.6 (23/1443)	1.7 (24/1422)	1.9 (25/1331)	1.2 (17/1432)	1.7 (25/1515)	1.7 (155/9351)
Sex (male), n (%)	10 (71.4)	18 (66.7)	17 (73.9)	17 (70.8)	19 (76)	15 (88.2)	17 (68)	113 (72.9)
Juvenile, n (%)	2 (14.3)	7 (25.9)	5 (21.7)	7 (29.2)	3 (12)	5 (29.4)	3 (12)	32 (20.6)
Adult, n (%)	12 (85.7)	20 (74.1)	18 (78.3)	17 (70.8)	22 (88)	12 (70.6)	22 (88)	123 (79.4)
Department, n (%)								
Neurology	4 (28.6)	5 (18.5)	6 (26.1)	5 (20.8)	5 (20.0)	2 (11.8)	3 (12)	30 (19.4)
General medicine	4 (28.6)	9 (33.3)	9 (39.1)	6 (25.0)	8 (32.0)	7 (41.2)	13 (52.0)	56 (36.1)
Intensive care unit	1 (7.1)	2 (7.4)	1 (4.3)	4 (16.7)	6 (24.0)	3 (17.6)	4 (16.0)	21 (13.5)
Pediatrics	0 (0)	6 (22.2)	2 (8.7)	2 (8.3)	3 (12.0)	2 (11.8)	2 (8.0)	17 (11.0)
Other	5 (35.7)	5 (18.5)	5 (21.7)	7 (29.2)	3 (12.0)	3 (17.6)	3 (12.0)	31 (20.0)

**Table 3:** The hospitalization data of the top 5 degenerative or genetic rare neurological diseases with most patients admitted to hospital in Guangdong Province of China (2016–2022)

	WD	ALS	PMD	ME	SMA
Number of patients	2634	2540	907	844	522
percentage of all enrolled RNDs (%)	28.2	27.2	9.7	9	5.6
Age, mean (SD), y	24.1 (13.0)	57.1 (11.6)	18.2 (17.4)	25.2 (16.9)	12.2 (13.9)
Sex (male), n (%)	1468 (55.7)	1599 (63.0)	788 (86.9)	502 (59.5)	320 (61.3)
Juvenile, n (%)	811 (30.8)	6 (0.2)	542 (60.0)	275 (32.6)	372 (71.3)
Adult, n (%)	1823 (69.2)	2534 (99.8)	365 (40.0)	569 (67.4)	150 (28.7)
Length of hospital stay, median (IQR)	12(7–16)	10 (7–15)	7 (3–12)	9 (5–14)	3(1–9)
Hospital admission	3578	3909	1045	1196	1174
Admission department, n (%)					
Neurology	2114 (59.1)	1617 (41.4)	188 (18.0)	720 (60.2)	315 (26.8)
Medicine	347 (9.7)	1239 (31.7)	373 (35.7)	92 (7.7)	60 (5.1)
Pediatrics	433 (12.1)	2 (0.0)	279 (26.7)	229 (19.1)	481 (41.0)
ICU	4 (0.1)	131 (3.4)	17 (1.6)	47 (3.9)	128 (10.9)
Others	680 (19.1)	920 (23.5)	188 (18.0)	108 (9.0)	190 (16.2)
Source of patients, n (%)					
District of the hospital	449 (12.5)	1577 (40.3)	337 (32.2)	391 (32.7)	270 (23.0)
The other district of local city	578 (16.2)	954 (24.4)	232 (22.2)	298 (24.9)	484 (41.2)
The other city of GD	1763 (49.3)	925 (23.7)	278 (26.6)	386 (32.3)	344 (29.3)
The other province	779 (21.8)	424 (10.8)	186 (17.8)	118 (9.9)	75 (6.4)
Others	9 (0.3)	29 (0.7)	12 (1.1)	2 (0.17)	1 (0.1)

RND, rare neurological disease; SD, standard deviation; IQR, interquartile range; ICU, intensive care unit; GD, Guangdong province

### Characteristics of ICU and death cases of RNDs

Altogether, there were 330 ICU cases recorded in the 7 years. The percentage of ICU cases among all included RND cases increased from 2.7% (28 patients), 2.9% (34 patients), 1.7% (24 patients), 2.7% (38 patients), 3.5% (46 patients), 3.8% (54 patients) to 7.0% (106 patients) in the 7-year period. Among these ICU cases, the percentage of juveniles grew annually from 10.7% (3/28), 17.7% (6/34), 16.7% (4/24), 29.0% (11/38), 28.3% (13/46), 44.4% (24/54) to 52.8% (56/106) (Fig. 1). Joinpoint regression demonstrated a significant upward trend of inpatient juvenile proportions in ICU patients with RNDs during the study period (APC = 28.42, *P* < 0.05, Fig. 1B), whereas no significant temporal change on proportions of ICU patients in all 20 RNDs was observed (Fig. 1B).

In total, 155 patients died, including 82 ALS patients, 26 PMD patients, 23 ME patients, 11 WD patients, 5 SMA patients, 2 PHD patients, 2 PMD patients, 2 X linked patients, 1 HD patient and 1 Kennedy patient. Annually, there were 14 deaths in 2016, 27 in 2017, 23 in 2018, 24 in 2019, 25 in 2020, 17 in 2021, and 25 in 2022; therefore, the number of annual deaths remained stable over the years. Among these 155 death cases, 123 were adults and 32 were juveniles. Male dominance was 72.9 (113/155) among the deaths. Most of these deaths were patients in the general medicine department (36.1%), neurology department (19.4%), and ICU department (13.5%) (Table 2).

### Burden of hospitalization

The total number of related hospital admissions was 13,678, with an average admission/year of 1.46. The majority of RND patients admitted to hospital were sent to neurology (45.3%), general medicine (20.7%), and pediatrics departments (13.3%). The percentage of RND patients who were admitted to neurology departments decreased from 52.2%, 46.8%, 50.4%, 50.0%, 43.5%, 42.8 to 37.4% over the 7 years. While admissions to pediatrics departments showed a tendency to increase from 6.9%, 11.7%, 10.9%, 8.6%, 14.2%, 13.3 to 21.5%. The number of RND patients from another province who were admitted to hospitals in GD showed a clear decline in 2022 compared with the average percentage (8.7% vs. 14.1%) (Table 1).

The median length of hospital stay reduced from 11 days, 10 days, 9 days, 9 days, 9 days, 9 days to 6 days over 7-year period. The majority of RND patients paid for their hospitalization via Urban Employee Basic Medical Insurance (UEBMI, 35.6%) and Urban Resident Basic Medical Insurance (URBMI, 21.1%). The percentage of fully self-funded patients decreased gradually from 29.1%, 29.4%, 24.8%, 19.0%, 16.6%, 16.7 to 8.8% over the study period (Table 1).

### Demographic and clinical characteristics of patients with the top five RNDs

**Fig. 2:**
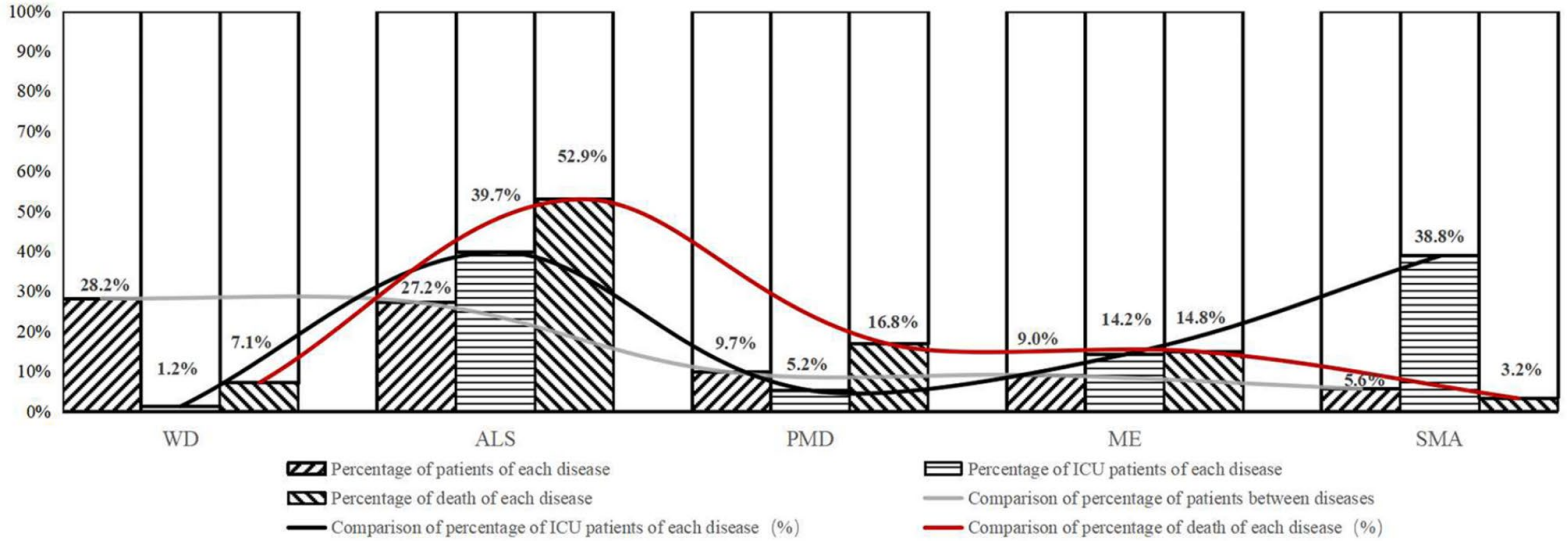
The percentage of patients, ICU patients and death of the 5 top rare neurological diseases with largest number of cases. The percentage of patients’ numbers compared with all recorded patients were 28.2% in WD, 27.2% in ALS, 9.7% in PMD, 9.0% in ME and 5.6% in SMA. That of ICU patients compared with all recorded ICU patients were 1.2%, 39.7%, 5.2%, 14.2% and 38.8% separately. The percentage of death were 7.1% in WD, 52.9% in ALS, 16.8% in PMD, 14.8% in ME and 3.2% in SMA. ICU, intensive care unit; WD, Wilson disease; ALS, amyotrophic lateral sclerosis; PMD, progressive muscular dystrophy; ME, mitochondrial encephalopathy; SMA, spinal muscular atrophy

The top 5 RNDs with the most cases in our study were WD, ALS, PMD, ME, and SMA, accounting for 79.7% (7447/9351) of all recorded cases. The percentages for the individual diseases were 28.2% (2634/9351, WD), 27.2% (2540/9351, ALS), 9.7% (907/9351, PMD), 9.0% (844/9351, ME), and 5.6% (522/9351, SMA) of the total patients (Table 3). The mean ages of the patients with the five diseases were 24.1 y, 57.1 y, 18.2 y, 25.2 y, and 12.2 y, respectively. The percentages of juveniles differed among the diseases, with 30.8% WD, 0.2% ALS, 60.0% PMD, 32.6% ME, and 71.3% SMA (Table 3). The proportions of each disease within total ICU cases were 1.2% (4/330) WD, 39.7% (131/330) ALS, 5.2% (17/330) PMD, 14.2% (47/330) ME, and 38.8% (128/330) SMA (Fig. 2). The proportions within total death cases were 7.1% (11/155) WD, 52.9% (82/155) ALS, 16.8% (26/155) MD, 14.8% (23/155) ME, and 3.2% (5/155) SMA (Fig. 2). Altogether, the top 5 RNDs accounted for 99.1% (327/330) of all ICU cases and 94.8% (147/155) of all death cases. Among these diseases, WD had the highest percentage (21.8%) of patients from other provinces treated in GD. Whereas most ALS patients (64.7%) were from local cities.

## Discussion

In this study, we demonstrated that the number of inpatient juveniles cases of 20 RNDs increased annually from 2016 to 2022 in Guangdong, a province that encompasses two-thirds of the South China population. Meanwhile, the incidences of juvenile ICU cases rose over the 7-year period. The growth in the inpatient health burden of RNDs was mainly evident in juveniles. The top five most common RNDs were WD, ALS, PMD, ME, and SMA, which also had the majority of death cases and nearly all ICU cases. To our knowledge, this is the first study to investigate the health burden of RNDs over such a long period in China.

As important forms of RD, RNDs comprised 5.8% and 13.4% of juvenile and adult RDs and accounted for 20.6% and 53.4% of juvenile and adult death cases respectively, in a study in Italy [9]. Nearly 82% of RD cases were registered in five clinical departments in China: neurology, endocrine, hematology, cardiovascular, and nephrology [4]. However, studies into the prevalence of RNDs in China are lacking. Our analysis of RNDs in GD reflects the situation in South China overall, as GD is the most significant economic province in China and includes two-thirds of the population of South China.

In a Hong Kong study that described the prevalence and health burden of all categories of RNDs from 2014 to 2018 [10], the proportion of adults was 86.4%, and the ratio of males to females was 1.12:1, compared to 74% and 1.76:1, respectively, in GD in our study. Thus, more male and juvenile cases were recorded in our study. The percentage of juveniles among all enrolled RNDs increased annually in GD. In accordance with this, the number of patients admitted to hospital pediatrics departments grew, while the number admitted to neurology departments decreased. Among the five RNDs with the most cases, we found that the percentages of juveniles among those with SMA (71.3%) and PMD (60%) were quite high. Furthermore, the percentage of juveniles increased annually among all ICU patients. Although the number of deaths was small among juveniles, the health burden of RNDs on juveniles was significantly heavier due to the larger population and comparatively more ICU cases. The tendency was more obvious in 2021 and 2022, which might have been due to the impact of COVID19 [19]. Getting access to healthcare during the pandemic became difficult due to RND patients and carers perceived risk of COVID infection and the strain on non-emergency healthcare resources. The early identification of RNDs is important to improve the prognosis in juveniles. Moreover, the transition of RNDs from the pediatrics department to the neurology department is crucial in hospitals without a pediatric neurologist. Although there was no significant temporal change on the proportions of ICU patients in all 20 RNDs, it still showed an increasing trend in 2021 and 2022. The reason might be similar with juvenile ICU RNDs.

RNDs tend to have a high prevalence and mortality rate compared with other RDs [8, 20]. Ratio of death was low and stable during these seven years which may show the solid quality of hospital care. The male dominance of mortality is also parallel with the male dominance overall (63%). We demonstrated that the top five RNDs (WD, ALS, PMD, ME, and SMA) accounted for 80% of patients with the 20 genetic RNDs, nearly all of the ICU cases, and the majority of death cases in this study. The prevalence of WD in 8 provinces of China was 2.85/100,000 from 2013 to 2016 [21]. In our research, WD accounted for the largest number of in-patient cases but the lowest percentage of ICU and death cases in GD. WD cases had the highest percentage of patients from other provinces, while the majority of ALS patients were from local cities. The number of ALS cases was also large, and there were extremely high death and ICU incidence rates. Although the number of SMA patients was relatively low, the incidence of SMA among ICU patients was high. Accurate diagnosis and high-quality treatment are important for all RNDs, especially those with large numbers of patients and a high health burden. Providing adequate resources for these diseases might maximize the benefits of RND management. The Chinese government has announced a series of policies to support the diagnosis and treatment of RDs, including fast-tracking orphan drugs, coverage by medical insurance, and disease registration [22]. In our study, we found that the percentage of fully self-funded RND patients significantly decreased from 29.1% in 2016 to 8.8% in 2022.

## Limitations

Our study has some limitations. First, this was a retrospective study that only included the front sheet data for inpatient cases in GD. Due to restrictions on information, we can’t enroll the outpatient RNDs data, which might include more comprehensive characteristics and reflect the health burden of RNDs more accurately. We will include more factors such as outpatient data, treatment strategy and hospitalization costs in the prospective study in the future. Second, our inpatient data can’t demonstrate the tendency of the total RND population. Higher RND hospital admissions might have alternative reasons, just like better access to hospital treatment, improved care pathways, and better recognition of the disease. Third, there are many other RNDs in addition to the 20 diseases analyzed in our study. We chose these 20 degenerative or genetic RNDs because they are listed in the Chinese First National List of RDs and might represent the situation in China.

## Conclusion

Our research analyzed the changing trend in the health burden of 20 RNDs from 2016 to 2022 in South China. The increase of the enrolled inpatient RND population and percentage of ICU patients, especially in juveniles, may demonstrate that there was an elevation in disease burden of juvenile RNDs in south China during these 7 years. In addition, the top five diseases accounted for nearly all ICU and death cases. Providing adequate resources for these diseases might maximize the overall benefits of RND management.

## Electronic supplementary material

Below is the link to the electronic supplementary material.

Supplementary Material 1

Supplementary Material 2

## Data Availability

The datasets used and/or analyzed during the current study are available from the corresponding author on reasonable request.
