# Supplementary material for: Incidence and health burden of 20 rare neurological diseases in South China from 2016 to 2022: a hospital-based observational study
Source: Orphanet J Rare Dis. 2025 Apr 8;20:163. doi: 10.1186/s13023-025-03704-5 (PMC11977943; doi:10.1186/s13023-025-03704-5)
Supplement: Supplementary file 1 — Supplementary Material 1 [file 13023_2025_3704_MOESM1_ESM.docx]

Supplementary Table 1. The number of cases for each of the 20 RNDs from 2016 to 2022

| RNDs | 2016 | 2017 | 2018 | 2019 | 2020 | 2021 | 2022 | Total |
| --- | --- | --- | --- | --- | --- | --- | --- | --- |
| WD | 342 | 399 | 437 | 420 | 332 | 400 | 304 | 2634 |
| ALS | 286 | 298 | 326 | 385 | 389 | 423 | 433 | 2540 |
| PMD | 86 | 126 | 183 | 119 | 117 | 139 | 137 | 907 |
| ME | 85 | 92 | 136 | 118 | 143 | 139 | 131 | 844 |
| SMA | 26 | 35 | 26 | 47 | 39 | 73 | 276 | 522 |
| HSP | 26 | 25 | 43 | 46 | 43 | 57 | 50 | 290 |
| PHD | 47 | 58 | 68 | 53 | 40 | 17 | 9 | 292 |
| CMT | 36 | 32 | 48 | 38 | 39 | 51 | 45 | 289 |
| SBMA | 23 | 23 | 36 | 28 | 22 | 38 | 25 | 195 |
| SCA | 23 | 20 | 36 | 33 | 23 | 6 | 1 | 142 |
| ALD | 13 | 16 | 17 | 24 | 22 | 25 | 18 | 135 |
| Dravet Syndrome | 6 | 16 | 24 | 19 | 20 | 14 | 9 | 108 |
| MMN | 16 | 14 | 30 | 23 | 19 | 0 | 0 | 102 |
| CADASIL | 0 | 0 | 17 | 31 | 38 | 0 | 0 | 86 |
| HD | 1 | 1 | 0 | 16 | 25 | 24 | 13 | 80 |
| Fabry | 1 | 2 | 2 | 5 | 5 | 14 | 50 | 79 |
| MD | 6 | 7 | 5 | 7 | 8 | 4 | 8 | 45 |
| LHON | 7 | 5 | 6 | 2 | 2 | 3 | 0 | 25 |
| CM | 3 | 1 | 1 | 3 | 4 | 1 | 5 | 18 |
| CMS | 1 | 4 | 2 | 5 | 1 | 4 | 1 | 18 |
| Total | 1034 | 1174 | 1443 | 1422 | 1331 | 1432 | 1515 | 9351 |

Amyotrophic lateral sclerosis (ALS), X-linked adrenoleukodystrophy (ALD), Charcot-Marie-Tooth Disease (CMT), Cerebral autosomal dominant arteriopathy with subcortical infarcts and leukoencephalopathy (CADASIL), Congenital myotonia (CM), Congenital myasthenic syndrome (CMS), Fabry disease, Hereditary spastic paraplegia (HSP), Huntington disease (HD), Leber hereditary optic neuropathy (LHON), Myotonic dystrophy (MD), Mitochondrial encephalopathy (ME), Multi-focal motor neuropathy (MMN), Primary hereditary dystonia (PHD), Progressive muscular dystrophy (PMD), Dravet syndrome, Spinal and bulbar muscular atrophy (SBMA), Spinal muscular atrophy (SMA), spinocerebellar ataxia (SCA) and Wilson disease (WD) .

Supplementary Table 2. The number of ICU cases for each of the 20 RNDs from 2016 to 2022

| RNDs | 2016 | 2017 | 2018 | 2019 | 2020 | 2021 | 2022 | Total |
| --- | --- | --- | --- | --- | --- | --- | --- | --- |
| ALS | 12 | 12 | 11 | 14 | 23 | 23 | 35 | 130 |
| SMA | 2 | 0 | 2 | 5 | 5 | 12 | 46 | 72 |
| ME | 7 | 7 | 4 | 9 | 11 | 13 | 9 | 60 |
| PMD | 6 | 3 | 2 | 4 | 1 | 2 | 4 | 22 |
| WD | 0 | 7 | 2 | 3 | 4 | 1 | 4 | 21 |
| Fabry | 0 | 0 | 1 | 0 | 0 | 0 | 5 | 6 |
| Darvet | 0 | 2 | 1 | 1 | 1 | 0 | 0 | 5 |
| ALD | 0 | 1 | 0 | 0 | 0 | 0 | 2 | 3 |
| MMN | 0 | 1 | 0 | 1 | 1 | 0 | 0 | 3 |
| SCA | 1 | 0 | 0 | 0 | 0 | 1 | 0 | 2 |
| PHD | 0 | 1 | 1 | 0 | 0 | 0 | 0 | 2 |
| HSP | 0 | 0 | 0 | 0 | 0 | 1 | 0 | 1 |
| CMT | 0 | 0 | 0 | 0 | 0 | 0 | 1 | 1 |
| CMS | 0 | 0 | 0 | 1 | 0 | 0 | 0 | 1 |
| MD | 0 | 0 | 0 | 0 | 0 | 1 | 0 | 1 |
| Total | 28 | 34 | 24 | 38 | 46 | 54 | 106 | 330 |

Amyotrophic lateral sclerosis (ALS), X-linked adrenoleukodystrophy (ALD), Charcot-Marie-Tooth Disease (CMT), Congenital myasthenic syndrome (CMS), Fabry disease, Hereditary spastic paraplegia (HSP), Myotonic dystrophy (MD), Mitochondrial encephalopathy (ME), Multi-focal motor neuropathy (MMN), Primary hereditary dystonia (PHD), Progressive muscular dystrophy (PMD), Dravet syndrome, Spinal muscular atrophy (SMA), Spinocerebellar ataxia (SCA) and Wilson disease (WD).

| RNDs | 2016 | | 2017 | 2018 | 2019 | 2020 | 2021 | 2022 | Total |
| --- | --- | --- | --- | --- | --- | --- | --- | --- | --- |
| WD | 72 | | 117 | 131 | 125 | 116 | 140 | 110 | 811 |
| PMD | | 53 | 72 | 108 | 65 | 64 | 93 | 87 | 542 |
| SMA | | 6 | 22 | 16 | 29 | 32 | 58 | 209 | 372 |
| ME | | 22 | 27 | 40 | 33 | 53 | 53 | 47 | 275 |
| Dravet Syndrome | | 4 | 16 | 20 | 17 | 18 | 13 | 9 | 97 |
| ALD | | 8 | 12 | 13 | 16 | 13 | 17 | 16 | 95 |
| CMT | | 6 | 7 | 13 | 12 | 10 | 13 | 11 | 72 |
| HSP | | 4 | 4 | 12 | 7 | 10 | 12 | 14 | 63 |
| PHD | | 4 | 10 | 9 | 7 | 7 | 2 | 2 | 41 |
| CMS | | 0 | 2 | 1 | 5 | 4 | 1 | 0 | 13 |
| Fabry | | 0 | 0 | 0 | 0 | 2 | 4 | 5 | 12 |
| LHON | | 4 | 1 | 5 | 1 | 1 | 0 | 0 | 12 |
| CM | | 0 | 0 | 1 | 2 | 3 | 0 | 3 | 9 |
| SCA | | 0 | 2 | 2 | 1 | 2 | 1 | 0 | 8 |
| ALS | | 0 | 0 | 2 | 0 | 1 | 2 | 1 | 6 |
| HD | | 0 | 0 | 0 | 0 | 1 | 0 | 1 | 2 |
| MMN | | 1 | 0 | 1 | 0 | 0 | 0 | 0 | 2 |
| MD | | 0 | 1 | 0 | 0 | 0 | 0 | 0 | 1 |
| CADASIL | | 0 | 0 | 0 | 0 | 0 | 0 | 0 | 0 |
| SBMA | | 0 | 0 | 0 | 0 | 0 | 0 | 0 | 0 |
| Total | | 184 | 293 | 374 | 320 | 337 | 409 | 515 | 2432 |

Supplementary Table 3. The number of pediatric cases for each of the 20 RNDs from 2016 to 2022.

Amyotrophic lateral sclerosis (ALS), X-linked adrenoleukodystrophy (ALD), Charcot-Marie-Tooth Disease (CMT), Cerebral autosomal dominant arteriopathy with subcortical infarcts and leukoencephalopathy (CADASIL), Congenital myotonia (CM), Congenital myasthenic syndrome (CMS), Fabry disease, Hereditary spastic paraplegia (HSP), Huntington disease (HD), Leber hereditary optic neuropathy (LHON), Myotonic dystrophy (MD), Mitochondrial encephalopathy (ME), Multi-focal motor neuropathy (MMN), Primary hereditary dystonia (PHD), Progressive muscular dystrophy (PMD), Dravet syndrome, Spinal and bulbar muscular atrophy (SBMA), Spinal muscular atrophy (SMA), spinocerebellar ataxia (SCA) and Wilson disease (WD) .

Supplementary Table 4. The number of 20 RNDs in all age groups from 2016 to 2022.

|  | 0-4 | 5-9 | 10-14 | 15-19 | 20-24 | 25-29 | 30-34 | 35-39 | 40-44 | 45-49 | 50-54 | 55-59 | 60-64 | 65-69 | 70-74 | 75-79 | 80-84 | 85- | Total |
| --- | --- | --- | --- | --- | --- | --- | --- | --- | --- | --- | --- | --- | --- | --- | --- | --- | --- | --- | --- |
| 2016 | 36 | 51 | 56 | 80 | 115 | 85 | 72 | 69 | 74 | 68 | 86 | 72 | 71 | 50 | 31 | 11 | 6 | 1 | 1034 |
| 2017 | 86 | 89 | 69 | 91 | 120 | 96 | 68 | 77 | 69 | 81 | 89 | 67 | 72 | 49 | 24 | 16 | 7 | 4 | 1174 |
| 2018 | 95 | 127 | 84 | 109 | 128 | 114 | 89 | 105 | 75 | 111 | 108 | 99 | 92 | 59 | 28 | 14 | 5 | 1 | 1443 |
| 2019 | 91 | 94 | 80 | 94 | 105 | 108 | 95 | 93 | 94 | 114 | 125 | 91 | 103 | 83 | 30 | 15 | 6 | 1 | 1422 |
| 2020 | 111 | 102 | 76 | 80 | 95 | 95 | 85 | 81 | 69 | 106 | 110 | 95 | 70 | 83 | 46 | 17 | 5 | 5 | 1331 |
| 2021 | 125 | 134 | 91 | 90 | 92 | 103 | 99 | 83 | 77 | 83 | 108 | 118 | 92 | 77 | 41 | 13 | 4 | 2 | 1432 |
| 2022 | 157 | 170 | 132 | 93 | 80 | 103 | 93 | 83 | 71 | 79 | 113 | 125 | 72 | 76 | 42 | 19 | 3 | 4 | 1515 |
| Total | 701 | 767 | 588 | 637 | 735 | 704 | 601 | 591 | 529 | 642 | 739 | 667 | 572 | 477 | 242 | 105 | 36 | 18 | 9351 |

Supplementary Table 5. The number of ICU cases of 20 RNDs in all age groups from 2016 to 2022.

|  | 0-4 | 5-9 | 10-14 | 15-19 | 20-24 | 25-29 | 30-34 | 35-39 | 40-44 | 45-49 | 50-54 | 55-59 | 60-64 | 65-69 | 70-74 | 75-79 | 80-84 | 85- | Total |
| --- | --- | --- | --- | --- | --- | --- | --- | --- | --- | --- | --- | --- | --- | --- | --- | --- | --- | --- | --- |
| 2016 | 0 | 0 | 1 | 3 | 3 | 1 | 1 | 1 | 3 | 3 | 2 | 4 | 3 | 2 | 0 | 0 | 1 | 0 | 28 |
| 2017 | 3 | 2 | 1 | 1 | 5 | 5 | 1 | 2 | 2 | 1 | 3 | 3 | 2 | 0 | 2 | 1 | 0 | 0 | 34 |
| 2018 | 2 | 1 | 1 | 1 | 2 | 3 | 0 | 1 | 0 | 2 | 2 | 4 | 3 | 0 | 1 | 1 | 0 | 0 | 24 |
| 2019 | 5 | 2 | 2 | 2 | 4 | 0 | 1 | 1 | 2 | 2 | 5 | 1 | 5 | 4 | 1 | 1 | 0 | 0 | 38 |
| 2020 | 7 | 2 | 3 | 3 | 3 | 2 | 2 | 1 | 1 | 3 | 7 | 4 | 1 | 4 | 1 | 2 | 0 | 0 | 46 |
| 2021 | 11 | 4 | 5 | 4 | 0 | 1 | 2 | 1 | 2 | 3 | 5 | 2 | 5 | 5 | 3 | 1 | 0 | 0 | 54 |
| 2022 | 22 | 19 | 8 | 8 | 4 | 2 | 2 | 5 | 2 | 4 | 5 | 13 | 4 | 5 | 2 | 1 | 0 | 0 | 106 |
| Total | 50 | 30 | 21 | 22 | 21 | 14 | 9 | 12 | 12 | 18 | 29 | 31 | 23 | 20 | 10 | 7 | 1 | 0 | 330 |
