# Supplementary material for: Incidence and health burden of 20 rare neurological diseases in South China from 2016 to 2022: a hospital-based observational study
Source: Orphanet J Rare Dis. 2025 Apr 8;20:163. doi: 10.1186/s13023-025-03704-5 (PMC11977943; doi:10.1186/s13023-025-03704-5)
Supplement: Supplementary file 2 — Supplementary Material 2 [file 13023_2025_3704_MOESM2_ESM.doc]

| Inpatient Medical Record Home PageMedical Payment Method Health Insurance Card Number Health Card Number Number of Hospitalizations： Number of Hospitalizations | | | | | | | | | | |
| --- | --- | --- | --- | --- | --- | --- | --- | --- | --- | --- |
| Name Former Name Gender: □ 1. Male 2. Female Date of Birth: Year / Month / Day Age: (Y/M/D)  Nationality Ethnicity Newborn Birth Weight (g) Newborn Admission Weight (g)  Place of Birth: Province / City (County) Native Place: Province / City (County)  ID Number Occupation Marital Status: □ 1. Single 2. Married 3. Widowed 4. Divorced 9. Other  Current Address Phone Postal Code  Household Address Postal Code  Employer & Address Work Phone Postal Code  Contact Person's Name Relationship Address Phone  Admission Route □ 1. Emergency 2. Outpatient 3. Transfer from another medical institution 9. Other  Source □ 1.Local Distric 2.Local City 3.Local Province 4.Other Province 5.Hong Kong, Macao, and Taiwan 6.International  Admission Time: Year Month Day Hour Admission Department  Confirmed Diagnosis Date: Year Month Day  Department Transfer:___ 1. Year Month Day Hour Transferred to Department:   1. Year Month Day Hour Transferred to Department: 2. Year Month Day Hour Transferred to Department:   Discharge Time: Year Month Day Hour Discharge Department  Hospital Bed Number: __ Actual Length of Stay (Days):  Outpatient/Emergency Diagnosis: Disease Code: Outpatient/Emergency Physician:  Admission Diagnosis: Disease Code: | | | | | | | | | | |
| Discharge Diagnosis | Disease Code | Condition at Admission | | | | Condition at Discharge | | | | |
|  |  | 1.Yes  2.Clinical Undetermined  3.Unknown  4..No | | | | 1.Cured  2.Improved  3. not recovered  4.death  5.others | | | | |
| Primary Diagnosis： |  |  |  |  |  |  |  |  |  |  |
| Other Diagnoses： |  |  |  |  |  |  |  |  |  |  |
|  |  |  |  |  |  |  |  |  |  |  |
|  |  |  |  |  |  |  |  |  |  |  |
|  |  |  |  |  |  |  |  |  |  |  |
|  |  |  |  |  |  |  |  |  |  |  |
|  |  |  |  |  |  |  |  |  |  |  |
|  |  |  |  |  |  |  |  |  |  |  |
| Medical Complications (Postoperative, Anesthesia) |  |  |  |  |  |  |  |  |  |  |
| Hospital Infections (Total Cases and Names) |  |  |  |  |  |  |  |  |  |  |
| Case Classification □ A. General B. Acute C. Complex D. Critical Rescue Attempts times  Successful Rescues times Clinical Pathway case □ 1. yes 2. no | | | | | | | | | | |
| External Causes of Injury or Poisoning Disease Code | | | | | | | | | | |
| Pathological Diagnosis Pathology Number Disease Code | | | | | | | | | | |
| Tumor Staging Type □ 1.P Pathological 2.C Clinical T □ 0/ 1/ 2/ 3/ 4 N □ 0/ 1 /2 /3 M □ 0/ 1 Tumor Staging | | | | | | | | | | |
| Malignant Tumor Differentiation Degree □ 1.Well Differentiated 2.Moderately Differentiated 3.Moderately-Poorly Differentiated  4.Poorly Differentiated 5.Undifferentiated 9.Uncertain or Not Applicable | | | | | | | | | | |
| Drug Allergy □1.no 2.yes, Allergic Drug： Autopsy for Deceased Patients □ 0.- 1. yes 2. no | | | | | | | | | | |
| Blood Type □ 1.A 2.B 3.O 4.AB 5.Unspecified 6.untested Rh □ 1.negative 2.positive 3.uncertain 4.not tested Blood Transfusion □ 1.yes 2.no  Transfusion Reaction □ 1.yes 2.no | | | | | | | | | | |
| Diagnosis Consistency Outpatient vs. Discharge□ Admission vs. Discharge□ Preoperative vs. Postoperative□  Clinical vs. Pathology□ Radiology vs. Pathology □ 0. Not Done 1.Consistent 2.Inconsistent 3.Uncertain | | | | | | | | | | |
| CT number PET CT number MR number X-ray number B-mode ultrasound | | | | | | | | | | |
| Medical Record Quality □1.A 2.B 3.C Quality Control Physician     Quality Control Nurse  Quality Control Date  year  month  day Responsible Nurse____ coder | | | | | | | | | | |
| Department Director or Deputy Director Physician: _______ Work ID____; Attending Physician_______ Work ID____ Resident Physician_______ Work ID____; Fellow Physician _______ Work ID____  Intern Physician_______ Work ID____; Postgraduate Intern Physician______ Work ID____ | | | | | | | | | | |

| Surgery  date | Surgery Name | Surgery Level | Surgery Risk Grade (NNIS) | Surgeon | | | Incision  /Healing | Elective Surgery | Anesthesia Type/ASA Grade | Anesthesiologist | Surgery Code |
| --- | --- | --- | --- | --- | --- | --- | --- | --- | --- | --- | --- |
|  |  |  |  | Surgeon | Assistant | Assistant |  |  |  |  |  |
|  |  |  |  |  |  |  |  |  |  |  |  |
|  |  |  |  |  |  |  |  |  |  |  |  |
|  |  |  |  |  |  |  |  |  |  |  |  |
|  |  |  |  |  |  |  |  |  |  |  |  |
|  |  |  |  |  |  |  |  |  |  |  |  |
|  |  |  |  |  |  |  |  |  |  |  |  |
| Operation Date | Procedure Name | | | | Operating Physician | | | Elective Surgery | Anesthesia Type/ASA Grade | Anesthesiologist | Operation Code |
|  |  |  |  |  | Surgeon | Assistant | Assistant |  |  |  |  |
|  |  | | | |  |  |  |  | / |  |  |
|  |  | | | |  |  |  |  | / |  |  |
|  |  | | | |  |  |  |  | / |  |  |
|  |  | | | |  |  |  |  | / |  |  |
|  |  | | | |  |  |  |  | / |  |  |
| Unplanned Return to Operating Room After Surgery □ 1. Yes 2. No Follow-up □ 1. Yes 2. No  Follow-up Period: ______ months | | | | | | | | | | | |
| Discharge Method □ 1. Discharged per Medical Advice 2. Transferred per Medical Advice, Receiving Medical Institution: ______ 3. Transferred to Community Health Service Center/Township Hospital per Medical Advice, Receiving Medical Institution: ______ 4. Discharged Against Medical Advice 5. Deceased 9. Others | | | | | | | | | | | |
| Planned Readmission Within 31 Days After Discharge □ 1. No □ 2. Yes, Purpose: | | | | | | | | | | | |
| Presence of Craniocerebral Injury:____ Coma Duration for Patients with Craniocerebral Injury: ____  Before Admission: ___ days ___ hours ___ minutes  After Admission: ___ days ___ hours ___ minutes | | | | | | | | | | | |

Obstetric Delivery Infant Record Form：

| Infant NO. | Gender | | | | | Delivery Outcome | | | | | Infant  weight  (g) | | | Infant Outcome | | | | | Respiration | | | | | Hospital Infection Count | | | Primary Hospital-acquired Infection Name | | | ICD  number | | | Resuscitation Attempts | | | Successful Resuscitation Attempts | | |  |
| --- | --- | --- | --- | --- | --- | --- | --- | --- | --- | --- | --- | --- | --- | --- | --- | --- | --- | --- | --- | --- | --- | --- | --- | --- | --- | --- | --- | --- | --- | --- | --- | --- | --- | --- | --- | --- | --- | --- | --- |
|  | M | | F | | 1.Live birth  2.Stillbirth  3.Fetal death | | | | |  | | | 1.Deceased  2.Transferred to another hospital  3.Discharged | | | | | 1.Natural  2.Grade I Asphyxia  3.Grade II Asphyxia | | | | |  | | |  | | |  | | |  | | |  | | |  | |
| 1 | |  | |  | | |  |  |  | | |  | | |  |  |  | | |  |  |  | | |  | | |  | | |  | | |  | | |  | | |
| 2 | |  | |  | | |  |  |  | | |  | | |  |  |  | | |  |  |  | | |  | | |  | | |  | | |  | | |  | | |
| 3 | |  | |  | | |  |  |  | | |  | | |  |  |  | | |  |  |  | | |  | | |  | | |  | | |  | | |  | | |
| 4 | |  | |  | | |  |  |  | | |  | | |  |  |  | | |  |  |  | | |  | | |  | | |  | | |  | | |  | | |

## Oncology Specialty Patient Treatment Record:

| 1. Radiotherapy Type：□1. Radical 2. Palliative 3. Adjuvant Plan: □1. Continuous 2. Intermittent 3. Segmented   Device: □ 1. Cobalt 2. Linear Accelerator 3. X-ray 4. Brachytherapy | | |
| --- | --- | --- |
| 1.Primary Lesion Dose (First, Repeat): CY/ Session/ Day: Treatment Period: Year / Month - Year / Month | | |
| 2.Regional Lymph Node Dose (First, Repeat) CY/ Session/ Day: Treatment Period: Year / Month - Year / Month | | |
| 3. Metastatic Lesion Dose: CY/ Session/ Day: Treatment Period: Year / Month - Year / Month | | |
| Ⅱ.Chemeotherapy Method: □ 1. Radical 2. Palliative 3. Neoadjuvant 4. Adjuvant 5. New Drug Trial 6. Other  Administration: □ 1. Systemic 2. Intra-arterial 3. Intrathoracic 4. Intraperitoneal 5. Intrathecal 6. Other | | |
| Date | Chemotherapy Regimen | Efficacy (Complete, Partial, Improvement, Stable, Progression, Not Determined) |
|  |  | CR、 PR、 MR、 S、 P、 NA |
|  |  | CR、 PR、 MR、 S、 P、 NA |
